# GrpE Immunization Protects Against *Ureaplasma urealyticum* Infection in BALB/C Mice

**DOI:** 10.3389/fimmu.2020.01495

**Published:** 2020-07-31

**Authors:** Yanhong Tang, Fangyi Guo, Aihua Lei, Jing Xiang, Pengqin Liu, Wenyou Ten, Guozhi Dai, Ranhui Li

**Affiliations:** ^1^Chenzhou Hospital Affiliated to University of South China, Hunan, China; ^2^Hunan Provincial Key Laboratory for Special Pathogens Prevention and Control, Institute of Pathogenic Biology, Pathogenic Biology Institute, Medical College, University of South China, Hunan, China; ^3^The First People's Hospital of Chenzhou, Hunan, China; ^4^The First People's Hospital of Huaihua, Hunan, China

**Keywords:** nucleotide exchange factor (GrpE), *Ureaplasma urealyticum*, immunogenicity, immune protection, BALB/c mice

## Abstract

Nucleotide exchange factor (GrpE), a highly conserved antigen, is rapidly expressed and upregulated when *Ureaplasma urealyticum* infects a host, which could act as a candidative vaccine if it can induce an anti-*U. urealyticum* immune reaction. Here, we evaluated the vaccine potential of recombinant GrpE protein adjuvanted by Freund's adjuvant (FA), to protect against *U. urealyticum* genital tract infection in a mouse model. After booster immunization in mice with FA, the GrpE can induced both humoral and cellular immune response after intramuscular injection into BALB/c mice. A strong humoral immune response was detected in the GrpE-immunized mice characterized by production of high titers of antigen-specific serum IgG (IgG1, IgG2a, and IgG3) antibodies. At the same time, the GrpE also induced a Th1-biased cytokine spectrum with high levels of IFN-γ and TNF-α after re-stimulation with immunogen GrpE *in vitro*, suggesting that GrpE could trigger the Th1 response when used for vaccination in the presence of FA. Although GrpE vaccination in the presence of a Th1-type adjuvant-induced had readily detectable Th1 responses, there wasn't increase inflammation in response to the infection. More importantly, the robust immune responses in mice after immunization with GrpE showed a significantly reduced *U. urealyticum* burden in cervical tissues. Histopathological analysis confirmed that tissues of GrpE-immunized BALB/c mice were protected against the pathological effects of *U. urealyticum* infection. In conclusion, this study preliminarily reveals GrpE protein as a promising new candidate vaccine for preventing *U. urealyticum* reproductive tract infection.

## Introduction

*Ureaplasma urealyticum*, one of the smallest self-replicating prokaryotic microorganisms, belongs to the Mollicutes class of bacteria and lacks a cell wall, although it evolved from Gram-positive ancestors (1). *U. urealyticum* is generally regarded as a low-virulent commensal, which commonly colonizes the adult genitourinary tract in humans (2). Adults are mainly infected through sexual contact, with the cervix being the main site of colonization in women. *U. urealyticum* colonization is associated with many diseases, including brain abscess, prostatitis, rheumatoid arthritis (3, 4), non-gonococcal urethritis, and hyperglycemia (5, 6). Maternal-fetal transmission seems to occur frequently, and intra-amniotic *U. urealyticum* infection may contribute to chorioamnionitis and preterm birth (7, 8). Individuals respond differently to *U. urealyticum* (6, 9, 10), with some able to clear an infection (as a result of a Th1/IFN-γ response), while others develop a chronic infection, and still others are susceptible to repeat infections. In particular, chronically asymptomatic infections can be frequent in women and may cause pelvic inflammation and sterility. Although *U. urealyticum* infections are often be cured by antibiotics, they can also be chronic, persistent, and antibiotic resistant (6, 11, 12). Therefore, the availability of a safe and effective vaccine would represent a much-needed and powerful means of protection from *U. urealyticum* infection and transmission.

Bacteria rapidly upregulate stress response-associated antigens as they infect their host, and therefore such antigens constitute attractive targets for vaccine development (13, 14). Heat shock proteins (HSPs) are molecular chaperones that perform essential cell functions under both normal and stress conditions and can be targets of the host immune system. In particular, HSP70 is recognized as an antigen by both the innate and adaptive immune responses of mammals and shows potent adjuvant activity (15). In eubacteria, the HSP70 system comprises DnaK-GrpE-DnaJ in *U. urealyticum*, and these three proteins are co-expressed. GrpE, a nucleotide exchange factor and cofactor of DnaK (HSP70) plays an important role in the survival of *U. urealyticum* during stresses such as heat-shock and hypoxia (16). It is expressed at equal levels as DnaK and plays a role in protein synthesis, folding, transportation, and degradation (17). Although the role of DnaK in the host immune response to bacterial infection has been widely studied, its cofactor GrpE has received relatively little attention and has not been reported on at all for *U. urealyticum* infections.

Preventive vaccines require target antigens to be expressed in the early stages of infection and to be recognized by the host immune system in order for host defense mechanisms to be rapidly activated (18, 19). When the *U. urealyticum* gene that encodes GrpE was sequenced and analyzed, using both the Smart BLAST database from NCBI and bioinformatics, it was predicted to be highly antigenic (Supplementary Figure 1). However, it is unknown whether GrpE can provide protection against *U. urealyticum* genital tract infection.

The present report represents the first study of the immunogenicity and potential protective efficacy of recombinant protein GrpE against *U. urealyticum* genital tract infection in a mouse model. We found that recombinant GrpE protein was quickly recognized by the host immune system, which was stimulated to produce high levels of IFN-γ. Following challenge with *U. urealyticum*, GrpE immunization reduced bacterial load and cervical inflammation in mice after genital tract infection. Therefore, our studies preliminarily proved that recombinant GrpE represents a promising new candidate vaccine against *U. urealyticum* infection.

## Materials and Methods

### U. urealyticum

The standard laboratory strain 8 (ATCC27618) of *U. urealyticum* was cultivated on *Mycoplasma* agar base supplemented with 10% fetal bovine serum (FBS; Gibco, USA), 0.3% urea and antibiotics at 37°C with 5% carbon dioxide for 24–48 h.

### Cloning, Expression, and Purification of Recombinant GrpE

The genome sequence of *U. urealyticum* was taken from GenBank. The full length GrpE sequence was amplified using PCR (polymerase chain reaction) with *U. urealyticum* genomic DNA as a template. The following primers were used: forward primer: 5′-CGC**CATATG**AGCAAAAACAACGAAAACATCAA-3′ (the *Nde I* site is underlined), reverse primer: 5′-CCG**GAGCTC**AATAAAGCGTTGAAATTGGTGGCG-3′ (the *Xho I* site is underlined).

The 657 bp PCR product obtained was purified, restriction digested, and inserted into expression vector pET28a (Qiagen, Shanghai, China) in frame with a His_6_ tag sequence at the N-terminal end (the His tag was an N-terminal fusion). The derivative, named pET28a-GrpE, was transferred into *E. coli* DH5α by CaCl_2_ transformation, and clones were selected on LB agar supplemented with 100 μg mL^−1^ kanamycin. The presence of the correct insert was then confirmed by double digestion and DNA sequencing. Finally, the plasmid pET28a-GrpE was transformed into *E. coli* BL21 (DE3).

For recombinant GrpE protein expression, transformed *E. coli* (pET28a-GrpE), and the vector control (pET28a in *E. coli* BL21 cells) were incubated in LB broth supplemented with 100 μg mL^−1^ kanamycin and 0.1 mM isopropy1-β-d-thiolgalactosidase (IPTG) at 37°C for 6 h. Bacterial cells were harvested by centrifugation at 4,000 × g for 15 min at 4°C, followed by resuspension of the pellets in lysis buffer [10 mM imidazole, 250 mM NaCl, 50 mM Tris-HCl (pH 7.8), 1% Triton X-100, 20% glycerol] and sonication. For protein purification, the GrpE protein in the supernatant was purified on a nickel-nitrilotriacetic acid (Ni-NTA) column, eluted with different imidazole concentrations. Endotoxins were removed by polymyxin B-agarose (Sigma-Aldrich, St. Louis, MO). Each step was evaluated by 12% SDS-PAGE using Coomassie brilliant blue staining and immunoblotting with an anti-His antibody (Lab Vision, Fremont, CA). Recombinant protein concentration was determined using a bicinchoninic acid protein assay kit (Pierce, Rockford, IL).

### Immunization of Mice

All animal procedures were approved by the Animal Welfare Committee of the University of South China and conducted according to institution regulations. We used a cohort of female, SPF, 6–8 weeks of age BALB/c mice (SJA Laboratory Animal Co., Hunan, China), with approval number SCXK (Hunan) 2016-0002. Three groups of mice (*n* = 18 in each group) were injected intramuscularly three times at 2-week intervals with either purified recombinant GrpE, phosphate-buffered saline (PBS), or Freund's adjuvant (FA). For the first immunization (day 0), 50 μg purified recombinant GrpE was emulsified in 100 μL Freund's complete adjuvant (FCA) (Sigma-Aldrich), and for the second and third immunizations (days 14 and 28) in 100 μL Freund's incomplete adjuvant (FIA). Mice in the PBS and FA groups were injected with 100 μL PBS or FA, respectively. Serum samples were collected by tail-vein bleeding 2 weeks after immunization.

### ELISA for Serum Antibody Levels

Two weeks after the last immunization, blood samples were collected from all mice by tail-vein bleeding prior to sacrifice. The serum was separated by centrifugation and stored at −20°C for further analysis. Serum samples were tested for antigen-specific antibody responses using ELISA methods described previously (20–22). Briefly, 96-well microplates were coated overnight at 4°C with purified recombinant GrpE, washed with 0.05% Tween 20 in PBS (PBST) and blocked with 5% nonfat milk in PBST at 37°C for 2 h. After further washes, a total of 100 μL of 1:1,000 dilutions of serum were added in duplicate to each well for 1 h at 37°C, respectively. After washing four times with PBST, 50 μL of horseradish peroxidase (HRP)-conjugated goat anti-mouse immunoglobulin G (IgG), IgG1, IgG2a, IgG3, and IgA, IgM antibodies (Protein Tech Group, Chicago, IL) was added to each serum well at 37°C for 1 h, respectively. After washing again, the plates were added 3, 3′, 5, 5′-tetramethylbenzidine substrate with 100 μL/well and incubated at room temperature for 15 min. The reaction was stopped by 100 μL of Stop Solution in each well, the absorbance (OD) at 450 nm was measured on a microplate reader (Thermo Lab systems, FI). The endpoint titer was considered to be the last serum dilution with readings higher than the mean+3 standard deviations of the negative control sera (23). Each experiment was repeated three times.

### Lymphocyte Proliferation Assay

Fourteen days after the final injection, spleens were harvested, and splenocytes were prepared as described previously (24). Cell viability was assessed using a colorimetric cell counting kit-8 (CCK-8) from Yi Yuan Biotechnologies (Guangzhou, China) (25). Single-cell suspensions (6 × 10^6^ cells) from adjuvant, PBS, and antigen-immunized mice were plated in 96-well plates and were stimulated with GrpE (10 μg mL^−1^) at 37°C, 5% CO_2_, and 95% humidity. After 44 h, 10 μL CCK-8 reagent was added to each well and incubated at 37°C for 4 h. The absorbance at 450 nm was measured and results were expressed as the proliferation index (PI), calculated based on the following formula: PI = [OD_450_ for stimulated cultures–OD_450_ for control group]/[OD_450_ for control group–OD_450_ for blank group].

### Quantification of Cytokine Expression in the Spleen

Spleen cells (6 × 10^6^/mL) were cultured in 24-well plates at 800 μL per well and stimulated with 10 μg mL^−1^ GrpE, incubated at 37°C in a 5% CO_2_ atmosphere for 48 h. Cell-free supernatants were harvested by centrifugation (6,000 rpm, 10 min) and stored at −80°C until use. Using an Essential Th1/Th2 Cytokine Panel (Invitrogen, e-Bioscience), four cytokines (IFN-γ, TNF-α, IL-4, and IL-10) were assayed by standard cytokine ELISA following the manufacturer's instructions. The sensitivities of the IFN-γ, TNF-α, IL-10, and IL-4 kits were in the ranges of 15–2,000, 8–1,000, 32–4,000, and 4–500 pg mL^−1^, respectively.

### Intracellular Cytokine Staining by Flow Cytometry

To assess the Th1 response, splenocytes were harvested for detection of intracellular interferon-gamma (IFN-γ) or interleukin (IL)-4. Cells (2 × 10^6^/well) were stimulated with PMA in 24-well plates at 37°C for 5 h. After washing, cells were first blocked with Fc Block (anti-mouse CD4^+^/CD8^+^, BD Biosciences) for 25 min. Then, after washing twice with PBS, cells were fixed and permeabilized for 25 min at 4°C using a cytofix/cytoperm kit (BD Biosciences) according to the manufacturer's instructions. Intracellular cytokines (IFN-γ, IL-4) were stained for 30 min in the dark. Finally, cells were washed with PBS and detected using a FACSverse flow cytometer and commercially available software (Flow Jo).

### Immunoblotting

We used GrpE-immunized mouse serum as the primary antibody. Two μL protein lysates were separated by SDS/PAGE, and then transferred to PVDF membranes (Millipore, Billerica, MA). Immunoblots were blocked with 5% skimmed milk in Tris-buffered saline/Tween-20 for 1 h at room temperature and probed with primary antibodies, including β-actin and anti-GrpE, and stored overnight at 4°C. Following three consecutive 5-min washes in TBS-T, blots were incubated with HRP-conjugated secondary antibody (goat anti-mouse IgG, Protein Tech Group, Chicago, IL) for 1 h at room temperature. After two washes in TBS-T and a final wash in TBS, blots were scanned using an enhanced chemiluminescence detection system (Amersham Pharmacia Biotech, CA), and quantification of antigen-antibody complexes was performed using Quantity One analysis software (Bio-Rad, Hercules, CA).

### Genital Tract Infection Challenge

Fourteen days after the last injection, each mouse was infected intravaginally (26) with 1 × 10^7^ colony forming units mL^−1^ *U. urealyticum* serotype 8 (50 μL) and the mice were kept supine for 1 min (27, 28). Seven days prior to infection, each mouse was injected with 0.5 mg estradiol benzoate subcutaneously in the neck once per week for 3 weeks to synchronize the estrus cycles and increase mouse susceptibility to *U. urealyticum* infection (29).

To identify mice infected with *U. urealyticum*, at days 0, 7, 14, and 21 after infection, an aseptic swab was inserted into the lower genital tract and uterus, rotated, and left in place for 1 min. Then, the swab specimens were washed into the liquid medium, which was then divided into two portions: one for *U. urealyticum* culture and determination of *U. urealyticum* concentration, and the other for PCR.

### Cytokine Measurement

To assess the levels of cytokines in the uterus and cervical secretions, IL-6, TNF-α, IL-1β, IL-10, IL-1α, IL-17a, monocyte chemotactic protein 1 (MCP-1), and IFN-γ were determined using a Multi-Analyte Flow Assay kit according to the manufacturer's instructions (Biolegend, San Diego, CA). After reference to a standard curve, the quantity of cytokines was reported as pg mL^−1^.

### Quantitative PCR

DNA preparation and PCR were performed as previously described (30–32). Quantitative real-time PCR was performed on genomic DNA (gDNA) extracted from *U. urealyticum*-challenged mouse tissues using a SYBR Green I assay. The *U. urealyticum* gDNA was quantified using primers *U.urealyticum*-1524R (5′-TTCCTGTGTTGCCCCTCAGTCT-3′) and *U. urealyticum*-1613F (5′-AAGGTCAAGGTATGGAAGATCCAA-3′), targeting 90 bp of the *U. urealyticum* urease gene (31, 33–35). The mouse gDNA was quantified using forward and reverse primers (5′-CCTTCCTTCTTGGGTATGGA-3′;5′-ACGGATGTCAACGTCACACT-3′, respectively), targeting 81 bp of the mouse β-actin gene (36). All primers were ordered from Integrated DNA Technologies (Sango Biotech, Shanghai, China). Quantitative real-time PCR reactions were performed in 20 μL reaction volumes according to the manufacturer's instructions (Qiagen). The amount of gDNA isolated was variable between tissue types but was normalized within each tissue type based on the lowest gDNA concentration obtained from the spectrophotometric measurements. A standard curve was created for the urease gene using a 10-fold serial dilution from 10^7^ to 10^1^ copies of linearized DNA with an efficiency of 99.30% and an R^2^ value of 0.9945. A standard curve was created for β-actin using a 2-fold serial dilution of mouse gDNA from 100 to 3.125 μg mL^−1^ with an efficiency of 99.05% and an R^2^ value of 0.9909. The assays were run on a Light Cycle 96 apparatus (Roche, Basel, Switzerland). PCR conditions for the urease gene were as follows: 95°C for 10 min, followed by 40 cycles of 95°C for 15 s, 58°C for 20 s, and 72°C for 20 s; subsequently, a melting curve was followed: 95°C for 10 s, 65°C for 60 s, and 97°C for 1 s. The PCR conditions for β-actin were as follows: 95°C for 10 min, followed by 40 cycles of 95°C for 15 s, 56°C for 20 s, and 72°C for 20 s; subsequently, a melting curve was followed: 95°C for 10 s, 65°C for 60 s, and 97°C for 1 s. Each assay was run with the following four controls: (1) no-template control; (2) no-amplification control (no Taq polymerase); (3) no-primer control; and (4) positive controls with a known concentration or copy number of mice gDNA or linearized urease gene plasmid DNA, respectively.

### Histopathology

All mice were anesthetized and euthanized through neck dislocation 21 days after infection. The whole reproductive tract was removed and fixed in formalin. After fixation, the tissue was treated according to standard procedures and embedded in paraffin wax. Sections were cut in the same area to include the uterine horn, fallopian tubes, and ovaries. The slices were placed on Superfrost glass and stained with hematoxylin and eosin (H&E). The slide was scanned using a 3D Hi-tech Panoramic Midi scanner (3D HISTECH Ltd., Budapest, Hungary) and evaluated by Case Viewer software (Nordic Biotite). The sections were evaluated by a pathologist blinded to the experimental treatments.

In addition, streptavidin-peroxidase (S-P) was used to detect *U. urealyticum* using an UltraSensitive^TM^ SP (mouse) IHC kit (Maixin, China). In short, the slices were treated with peroxisome blocking solution to inhibit background staining. For reproductive tract tissue, we used mouse anti-*U. urealyticum* as the first antibody, biotin-labeled goat anti-mouse (Service Bio, G1210-2-A, China) as the second antibody, and chromosome DAB solution as the substrate. Hematoxylin was used to double dye the slide. Normal mouse serum was used as a negative control.

### Statistical Analysis

All analyses were repeated at least two times with consistent results. The levels of significance for comparisons between samples were determined by analysis of variance (ANOVA) with the Student-Newman-Kaul's test, or by Student's *t*-test and nonlinear regression with an extra sum-of-squares F-test to investigate any apparent differences between the test and control groups using statistical software (GraphPad Prism, version 7; San Diego, CA). Results are expressed as means. Values of ^*^P < 0.05, ^**^P < 0.01, and ^***^P < 0.001 were considered statistically significant.

## Results

### Expression and Purification of Recombinant GrpE

**Figure 1:**
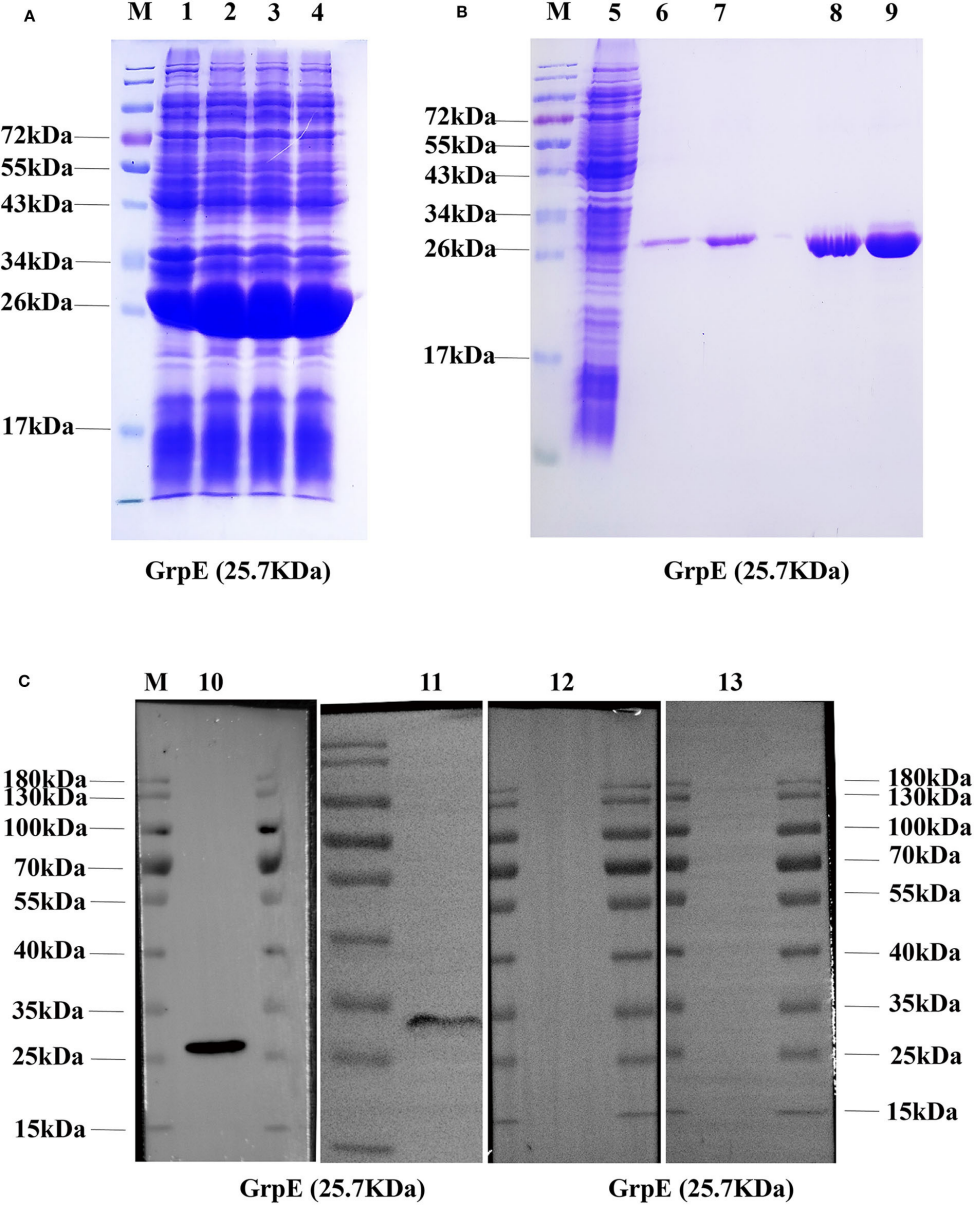
Production of recombinant GrpE protein: **(A)** recombinant GrpE was successfully produced in *E. coli* BL21, after induction with 0.1 mM IPTG for 6 h, and identified by 12% SDS-PAGE (M, molecular weight markers; lanes 1–4, induced); **(B)** 12% SDS-PAGE analysis of GrpE purified using a Ni-nitrilotriacetic acid (NTA) column (M, molecular weight markers; lanes 5, uninduced; lanes 6–9, purified GrpE by eluents with different concentrations of imidazole: 70, 90, 150, 200 mM); (**C**, Lane 10) Western blot analysis of purified GrpE using mouse anti-His antibodies. (**C**, Lane 11) GrpE identified by antibodies in GrpE-immunized mouse serum. (**C**, Lane 12, 13) FA- and PBS-group sera.

For the purification of recombinant GrpE, the protein was expressed with a histidine tag in *E. coli* BL21 (Figure 1A). The recombinant protein GrpE was eluted with different concentrations of imidazole. SDS-PAGE results showed that the purified protein GrpE was obtained at a concentration of 200 mM imidazole and revealed the recombinant protein to have the predicted molecular weight of about 25.7 kDa (Figure 1B). Protein purification was confirmed by immunoblotting with an anti-His antibody (Figure 1C), and this purified protein was used in the following experiments.

### Immunization of Mice With GrpE

**Figure 2:**
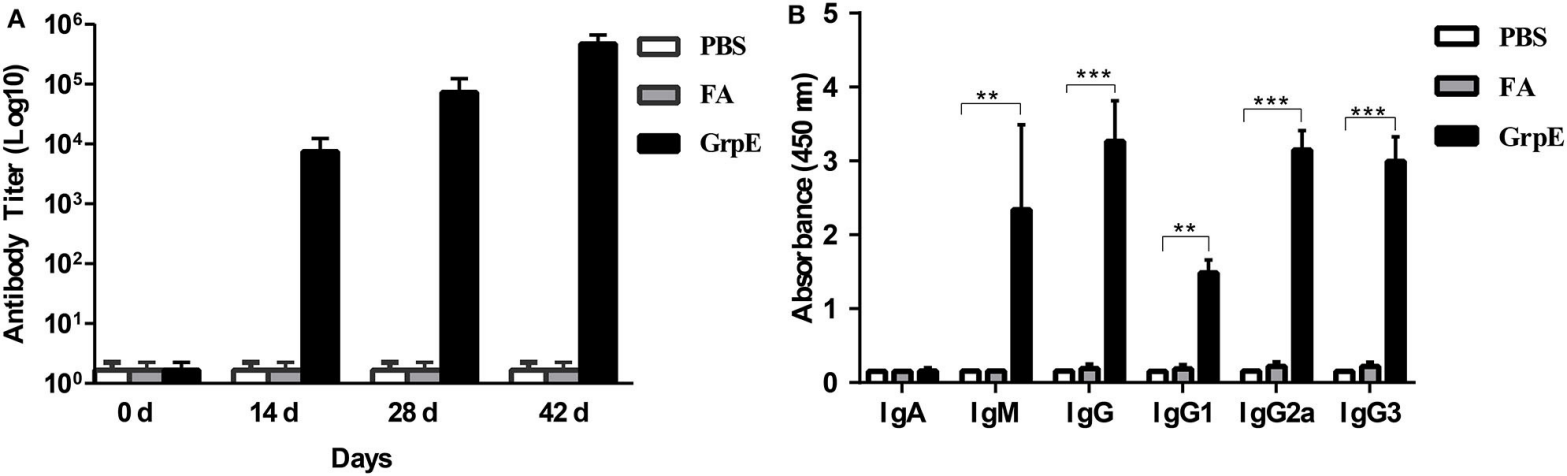
Effect of GrpE vaccination on Ig antibody production, including IgG subclasses. Three groups of BALB/c mice were immunized intramuscularly with PBS, FA or GrpE. Six mice from each group were sacrificed and sera were separated on day 14 after the last injection. Specific antibody levels in sera were assessed by ELISA: **(A)** anti-GrpE antibody titer; **(B)** GrpE-specific antibody levels and IgG subclass levels. Each bar indicates the mean ± SD of triplicates from six mice per group. *t*-test, ***P < 0.001, **P < 0.01.

To determine whether GrpE was able to induce an antibody response in mouse, 14 days after the final booster injection, an ELISA was performed to measure the level of GrpE-specific antibodies in sera. Specific antibodies were already present in GrpE-immunized mice on day 14, and antibody titers against the protein increased gradually thereafter, reaching maximum levels by day 42 (Figure 2A). The results clearly show that intramuscular injection of recombinant GrpE into BALB/c mice generates a specific antibody response.

Previous studies illustrated that IgM and IgG perform protective roles in the early and late stages of bacterial infection (37). Consequently, IgG, IgA, and IgM in the serum samples, which were collected from immunized mice 14 days after the last vaccination, were assessed by ELISA to determine the levels of these antibodies. As shown in Figure 2B, the GrpE vaccination group produced significantly higher levels of IgG and IgM antibodies than the FA and PBS control groups (*P* < 0.001), but there was no significant difference in IgA level. To examine the specific type of antibody response induced by GrpE, we determined the levels of IgG subclass antibodies (IgG1, IgG2a, and IgG3) in serum and found the levels of the serum IgG subclass antibodies (IgG1, IgG2a, and IgG3) induced in the recombinant antigen GrpE group were also significantly higher than those induced in the FA and PBS control groups (*P* < 0.01) (Figure 2B). The responses were indicative of a Th1 response with IgG2a and IgG3 antibodies and is as easy to detect as IgG1 antibodies.

### GrpE-Induced Lymphocyte Proliferation Response

To investigate whether vaccination of mice with GrpE stimulates a proliferative response in lymphocytes, experiments were carried out with splenic lymphocytes from all three groups of mice. Suspensions of splenocytes from mice immunized with GrpE showed an increase in lymphocyte proliferation, with significant differences to the PBS and FA groups (Supplementary Figure 3, *P* < 0.01).

### GrpE Immunization Predominantly Stimulates the Th1 Immune Response

**Figure 3:**
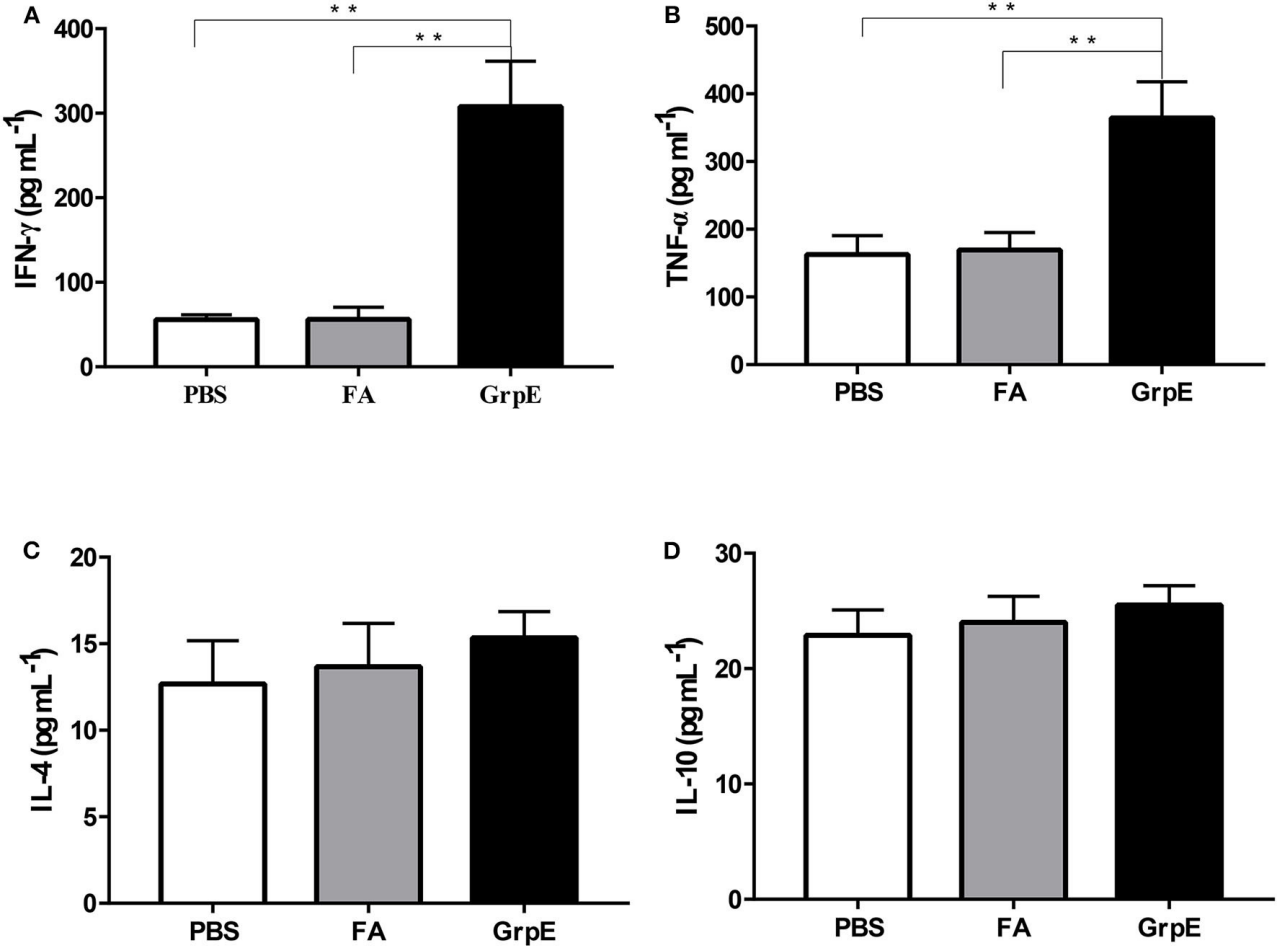
Intramuscular immunization with GrpE predominantly induces a Th1 immune response. Fourteen days after the last injection, the spleen was collected from each group (six mice in each group) and used to prepare a spleen cell suspension, which was stimulated *in vitro* with 10 μg GrpE at 37°C in a CO_2_ incubator for 48 h. The production of IFN-γ **(A)**, TNF-α **(B)**, IL-4 **(C)**, and IL-10 **(D)** in spleen cell culture was measured by ELISA. **P < 0.01 vs. PBS and FA groups analyzed by ANOVA.

To determine the type of cell response in GrpE-immunized mice, spleen cell culture medium was collected after stimulation and cytokine levels were measured by ELISA. The spleen cells of GrpE-immunized mice exhibited significantly higher levels of Th1 cell-related cytokines TNF-α and IFN-γ (Figures 3A,B, *P* < 0.01), whereas the Th2-related cytokines IL-4 and IL-10 showed no significant difference compared with PBS and FA groups (Figures 3C,D, *P* > 0.05). Taken together, these results suggest that the immunogenicity of GrpE is based on its ability to induce a protective T-cell response, and in particular a Th1 polarization response.

**Figure 4:**
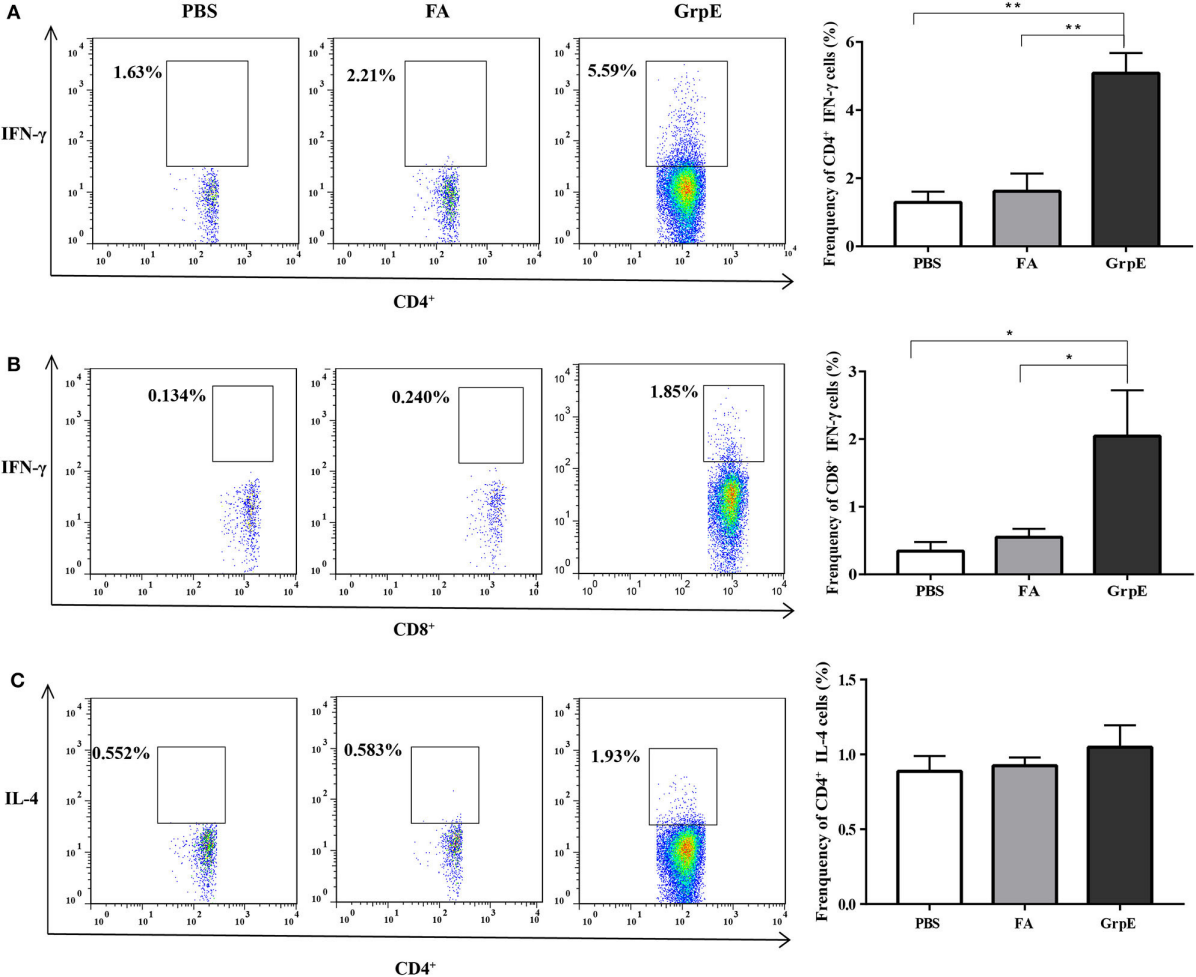
The generation of cytokines by T cells in spleen cell suspension measured by multi-parameter flow cytometry. Data were collected using a FACSverse flow cytometer and then analyzed by FlowJo software: **(A)** IFN-γ produced by CD4^+^ T cells; **(B)** IFN-γ produced by CD8^+^ T cells; **(C)** IL-4 produced by CD4^+^ T cells. *P < 0.05, **P < 0.01 vs. PBS and FA groups analyzed by ANOVA. Six mice in each group.

Although both CD4^+^ and CD8^+^ T cells are necessary to protect against *U. urealyticum* infection, we can monitor host immune system recognition by measuring levels of IFN-γ, the most important CD4^+^ Th1-related cytokine involved in the early immune response against this pathogen. Therefore, we investigated the production of IFN-γ by flow cytometry prior to *U. urealyticum* challenge. The results show that the frequency of CD4^+^ and CD8^+^ T cells producing IFN-γ was significantly increased in the spleens of the GrpE-immunized group, compared with controls (Figure 4A, *P* < 0.01; Figure 4B, *P* < 0.05). In contrast, there was no significant difference in IL-4 production between the GrpE-immunized and control groups (Figure 4C, *P* > 0.05).

### Immunization With Recombinant GrpE Protein Rrduces Colonization of *U. urealyticum* in the Cervix

**Figure 5:**
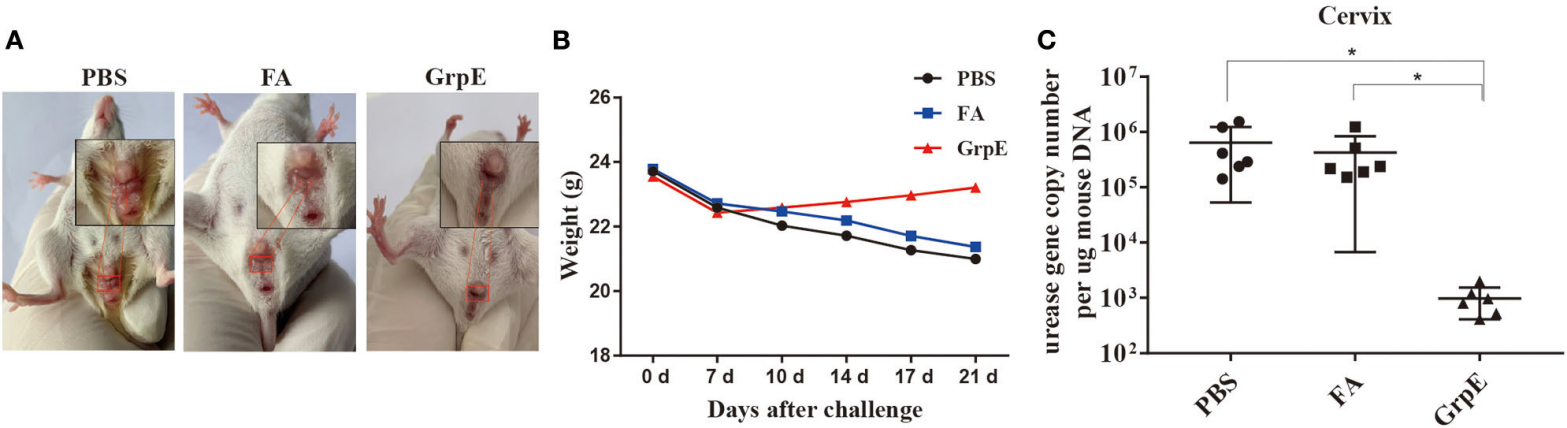
Immunization with recombinant GrpE protein reduces colonization of the cervix by *U. urealyticum*. Appearance of the vagina in mice after 21 days of infection **(A)**; change in body weight of BALB/c mice inoculated with *U. urealyticum*, recorded on day 0, 7, 10, 14, 17, and 21 **(B)**; *U. urealyticum* burden in control animals (PBS and FA groups) (*N* = 6) and animals immunized with recombinant GrpE protein (*N* = 6) measured by quantitative PCR of the urease gene concentration in diseased cervical tissue **(C)**. The Mann-Whitney test was used to normalize the results to the mouse gDNA concentration. Data points correspond to six samples taken from each animal. The horizontal line represents the average value (*P < 0.05).

To study the protective effect of GrpE immunization, immune BALB/c mice, together with the PBS and FA control groups, were infected with *U. urealyticum*. Analysis of microbial cultures and PCR results of vaginal and cervical secretions were positive for *U. urealyticum* (Supplementary Table 1), indicating successful infection. In addition, 1 week after infection, all mice showed vaginal peripheral hair loss, increased secretions, redness, and loss of appetite, but the symptoms in the GrpE group were relatively mild (Figure 5A). We also observed that, after challenge with *U. urealyticum*, mice showed a general loss of hair and weight, and consumed less food and water. However, while the PBS and FA groups continued to lose weight, the GrpE-immunized group gradually began to recover 1 week after the infection (Figure 5B).

To confirm whether GrpE immunization conferred protection against *U. urealyticum* at the colonization site (cervix), the burden of *U. urealyticum* in the lesion site was evaluated by quantitative PCR method on day 21 after inoculation. The results show that the level of *U. urealyticum* in the primary lesion site was significantly lower in GrpE-immunized mice than in controls (Figure 5C, *P* < 0.05). *U. urealyticum* was not detected in isolated liver, kidney, spleen, and lung tissues (data not shown).

### Inflammatory Response in Cervix Is Mitigated by GrpE Immunization

**Figure 6:**
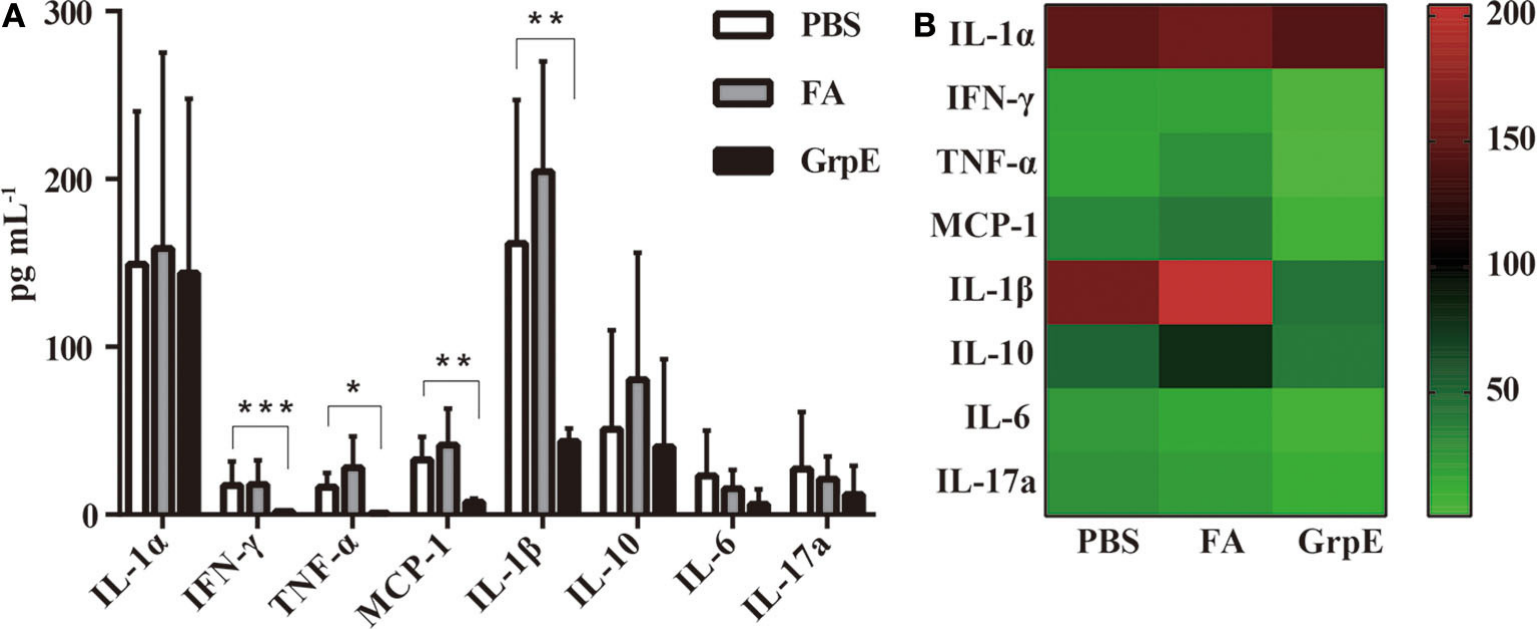
Cytokine levels in cervical tissue of mice challenged with *U. urealyticum* subsequent to immunization. A multi-analyte flow assay kit was used to detect IL-6, TNF-α, IL-1β, IL-10, IL-1α, IL-17a, MCP-1, and IFN-γ in the culture medium of cervical homogenates after *U. urealyticum* infection in mice: **(A)** Each bar represents the mean (± SD) cytokine level (pg ml^−1^) in cervical tissue homogenate of six mice in each group in three independent experiments. By ANOVA, *P < 0.05; **P < 0.01; ***P < 0.001. **(B)** Cluster analysis of inflammatory factors, each value representing the sample mean of each group.

A candidate vaccine must meet two criteria: (1) it must stimulate a protective immune response, and (2) it must eliminate the uncontrolled inflammation that causes pathological changes during infection. The second of these was assessed with a multi-analyte flow assay kit to determine the levels of IL-6, TNF-α, IL-1β, IL-10, IL-1α, IL-17a, MCP-1, and IFN-γ in the supernatant of cervical tissue homogenate of GrpE-immunized mice and control groups after vaginal infection. As shown in Figure 6A, there was no significant difference in IL-17a, IL-6, IL-10, and IL-1α levels between GrpE-immunized mice and control groups. However, the levels of IFN-γ (*P* < 0.001), TNF-α (*P* < 0.05), MCP-1 (*P* < 0.01), and IL-1β (*P* < 0.01) in the cervical cervix of the GrpE-immunized group were significantly lower than those of the controls. As shown in Figure 6B, a heat map analysis of the original data, the color shade and the gradual change from green to red indicate increased levels of cytokine secretion, the results show that IL-1α levels between GrpE-immunized mice and control groups are similar, IL-1β levels in control groups are obviously red, and green in the GrpE-immunized group.

### Immunization With GrpE Significantly Reduces Cervical Lesions

**Figure 7:**
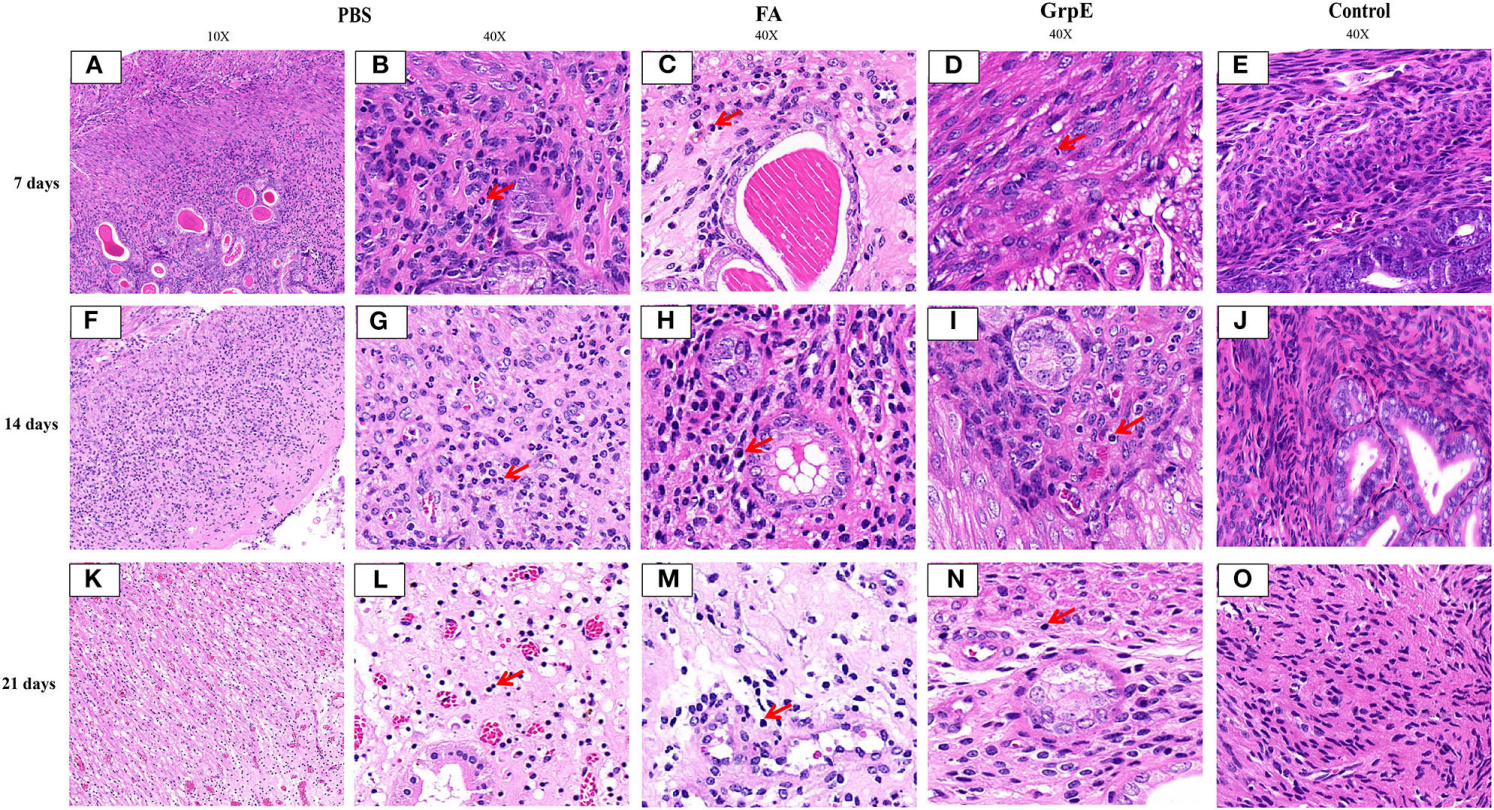
Pathological lesions of mouse cervical tissue after *U. urealyticum* challenge. Cervical tissue of PBS **(A,B,F,G,K,L)**, FA **(C,H,M)**, control **(E,J,O)**, or GrpE- immunized **(D,I,N)** mice was sectioned and stained with H&E on day 7, 14, and 21 after challenge with *U. urealyticum*. Arrows indicate inflammatory cells.

Finally, the protective effect of the recombinant antigen GrpE was further evaluated by comparing the inflammatory pathology of the mouse genital tract. *U. urealyticum* infection caused severe pathological damage to cervical tissues in the PBS (Figures 7A,B,F,G,K,L) and FA (Figures 7C,H,M) groups. Moreover, acute inflammation (a large number of polymorphonuclear leukocytes) was observed on day 7 after infection, which was converted to chronic inflammation by day 21 (mass lymphocytes) (Figures 7A,B,F,G,K,L,C,H,M; The red arrow in the figure). In contrast, the inflammatory infiltration in cervical tissues of GrpE-immunized mice was significantly decreased (Figures 7D,I,N). Compared with negative control animals (Figures 7E,J,O), the structure of gland and cervical tissue could clearly be identified, its integrity was maintained and fewer pathological features, such as glandular dilatation, increased glandular secretions, hemorrhage, and inflammatory cell infiltration were observed.

**Figure 8:**
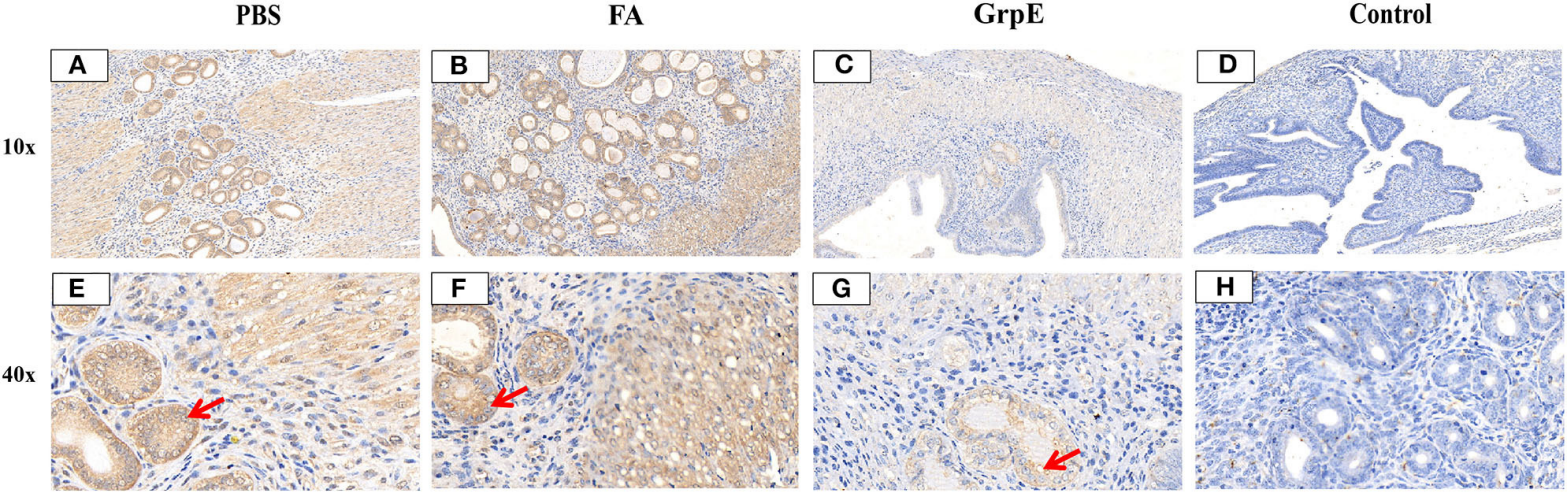
*U. urealyticum* load and pathological changes in lung tissue assessed by S-P immunohistochemistry. Cervical tissue of PBS **(A,E)**, FA **(B,F)**, and **(C,G)** GrpE-immunized mice was sectioned and stained with S-P immunohistochemistry 21 days after infection with *U. urealyticum* and the control group **(D,H)** was uninfected with *U. urealyticum*. The UltraSensitive™ SP (mouse) IHC kit, with a mouse anti-*U. urealyticum* first antibody (Provided by Pathogenic biology Institute, University of South China, Hunan, China) was used to detect *U. urealyticum* inclusion. Areas stained brown (red arrows) in the nuclei of cervical tissue cells contain a *U. urealyticum* inclusion.

Evaluation of cervical tissue sections 21 days after infection by S-P immunohistochemistry showed that the mice immunized with recombinant GrpE protein presented nearly normal cervical tissue compared with control group (Figures 8D,H) and fewer inflammatory cells in the cervical tissue (Figures 8C,G), compared to the FA- (Figures 8B,F) and PBS- (Figures 8A,E) immunized mice. In addition, the *U. urealyticum* load (brown; red arrow) in GrpE-immunized mice was significantly lower than in the PBS and FA groups (Figures 8A,B,E,F), where *U. urealyticum* was present in glands, interstitial cells, and cytoplasm. In general, no inflammation was detected in the fallopian tubes or ovarian sacs, while mild-to-moderate inflammatory infiltration was seen in the vagina and bladder.

## Discussion

In general, an effective antigen target for the rational design of a vaccine against *U. urealyticum* should meet the following requirements: it should be expressed constitutively; it should be recognized by the immune system at an early stage of infection *in vivo*; it should induce a Th1-biased immune response; and it should prevent *U. urealyticum* infection. In this study, we assessed *U. urealyticum* GrpE as a candidate vaccine and asked whether it could meet the above criteria and allow the host to effectively resist an attack by *U. urealyticum*.

Recombinant GrpE protein was successfully expressed in *E. coli* BL21 and purified by His-tag affinity chromatography. The size of the recombinant GrpE was slightly larger than predicted, probably due either to a difference in protein modification in the *E. coli* expression system or to the His-tag at the N-terminus or other molecules binding to the recombinant protein during the expression and purification of GrpE recombinant protein. Immunization of mice with recombinant GrpE induced the production of GrpE-specific antibodies as shown by immunoblotting.

Helper T cells play a vital role in the immune response that follows vaccination against a particular pathogen. They can be divided into two subsets, Th1 and Th2, which govern different aspect of the IgG response, namely IgG2a and IgG1 production, respectively (38, 39). After mice were immunized with recombinant GrpE protein with the presence of FCA, it showed high titers of serum anti-GrpE total antibody IgG, IgG1, IgG2a, and IgG3. Here, our results showed that the recombinant GrpE protein mainly induces Th1 response of higher IgG2a and IgG3 antibodies level, and is as easy to detect as IgG1 antibodies (Figure 2B).

These results suggest that recombinant GrpE protein can produce a humoral immune response that should prevent microbial attachment to tissue surfaces. Th1 and Th2 immune responses can be distinguished according to the type of cytokine secreted. While activated Th1 cells are responsible for secretion of IFN-γ and TNF-α, activated Th2 cells secrete IL-4 and IL-10 (40, 41). In the spleen cells of mice immunized with recombinant GrpE, IFN-γ and TNF-α levels increased significantly, but IL-4 and IL-10 levels did not differ from those of controls (Figure 3), indicating that GrpE antigen was rapidly recognized by the host immune system and may trigger a Th1-dominated immune response.

FCA is one of the most effective experimental adjuvants, that containing heat-killed and dried *Mycobacterium tuberculosis* and mineral oil, which was widely used to boost the immune response to a foreign antigen. The FCA can induce activation of the immune system non-specifically and entrap antigens in a water-in-oil emulsion, which can localizes antigens for release slowly, providing the chance to encounter antigen-presenting cells (42–45). Our results show the levels of antibodies and TNF-α and IFN-γ produced in the FA group were slightly higher than those of the PBS group, but there was no difference compared with the PBS group. The FA group may induce a very weak non-specific Th1 type response, but after the *U. urealyticum* challenge, the FA group's and the PBS group's cervical tissue *U. urealyticum* load situation and degree of pathological changes in cervical tissue were similar (see Figures 7, 8), indicating that this weak non-specific Th1-type response did not against *U. urealyticum* infection and cannot protect the body from *U. urealyticum* colonization. Our results also show GrpE vaccination in the presence of FCA could induce readily detectable Th1 responses; however, there wasn't increased inflammation in response to the infection or reduced pathological changes of the infected site. FCA are known to enhance the immunogenicity of the immunogen, play an immune regulation role, and increase the immune effect of the antigen, which can modulate immune cells and enhance humoral and cellular immune responses in the host (22, 46, 47). So we conclude that the GrpE protein in the presence of FCA may induce protective Th1 immune response in mice and be considered as an effective vaccine against *U. urealyticum* infection.

Based on these results, we evaluated the protective effect of GrpE immunization against the *U. urealyticum* strain. A previous study used BALB/c mice to study *U. urealyticum* serotype 8 infection (48) and showed that estradiol treatment is necessary for the successful establishment of a *U. urealyticum* infection model (28, 48). In the present study, the rate of *U. urealyticum* infection was up to 100% in PBS and FA groups, while the rate of solid culture of *U. urealyticum* from cervical secretions decreased after 14 days, which might reflect clearance of the pathogen (49). In addition, immunization with GrpE significantly reduced the *U. urealyticum* load and the extent of inflammation in the cervical tissue of infected mice. The relative decrease in *U. urealyticum* DNA concentration in the cervix in immunized animals was particularly significant. Nevertheless, despite the apparent clearance of *U. urealyticum* by 14 days post-inoculation, there were persistent abnormalities of the cervical epithelium and chronic inflammation from 14 to 21 days post-inoculation. Taken together, however, the results demonstrate that GrpE may be a promising vaccine candidate against *U. urealyticum*.

*In vitro* and *in vivo* experiments and clinical trials have shown that IFN-γ and TNF-α play a vital role in the elimination of *U. urealyticum* (50, 51), and the colonization of the cervix by *U. urealyticum* is associated with an increase in the concentrations of some pro-inflammatory or inflammatory factors, that is, IL-6, TNF-α, IL-1β, IL-10, IL-1α, IL-17a, MCP-1, and IFN-γ (38, 49, 52). TNF-α, which is the earliest-secreted cytokine in the inflammatory response, is mainly derived from mononuclear macrophages; it induces chemokine production, promotes the expression of adhesion molecules in epithelial cells and lymphocytes, and recruits inflammatory cells to the inflammation region (53). When inflammation occurs in the body, IFN-γ secreted by CD4^+^- and CD8^+^-positive cells immediately recruits immune cells to the infected site to clear the pathogen and to prevent it aggravating lesions and spreading (54). In this paper, we show that high levels of IFN-γ and TNF-α were produced in mice after immunization with GrpE, but the expression level at inflammatory sites was significantly decreased after *U. urealyticum* infection, demonstrating that IFN-γ and TNF-α can stimulate macrophages to increase their bactericidal activity and to limit the multiplication and spread of *U. urealyticum* (55, 56); TNF-α can also delay any related pathogenesis (57).

There were some limitations in our study. Thus, our sample size was rather small, which means that the statistical data may not be generally representative. At the very least, however, this study can guide future experiments in setting an adequate sample size. Our findings may have limited relevance to clinical disease in humans, since animal models do not precisely replicate the conditions in humans. *U. urealyticum* infection may change from an initial acute phase to a persistent, chronic form. Previously reported (48) that *U. urealyticum* can colonize the vagina for 163 days. During this period, the immunity of the body is low, or the microbial environment in the body changes, if the bacterial load in the lower reproductive tract is not completely cleared after the initial infection, the persistence of *U. urealyticum* in the organs will be a negative factor, and it is likely to infect the upper reproductive tract, liver, kidney, spleen, lung, intestine, and even brain, leading to serious inflammatory damage. The time interval (3 days) between *U. urealyticum* infection in mice and the first sampling point was rather long, and the lack of an earlier time point made it difficult to elucidate the temporal progress of the body's inflammatory responses as the microorganisms spread into the uterus and upper genital tract. Therefore, further research is needed to solve these problems.

Hartley et al. (58) questioned the protective efficacy of HSPs against microbial infection. These authors observed that the protective effect of HSP60 immunization against *Francisella tularensis*, the causative agent of tularemia, was due to the presence of contaminating lipopolysaccharide (LPS) in HSP60 preparations. In our study, the recombinant GrpE protein derived from *U. urealyticum* was cloned and expressed in a heterogenic host (*E. coli* BL21) and then purified by Ni-NTA affinity chromatography. It is therefore possible that the recombinant protein may contain a certain amount of LPS from *E. coli* cell walls, but since *U. urealyticum* itself has no cell wall, this is unlikely to be responsible for the protection given by GrpE immunization. Therefore, we conclude that the preliminarily protective effects observed in this study can be attributed to the recombinant GrpE protein of *U. urealyticum*.

## Conclusion

GrpE is recognized by the immune system at an early phase of infection and has the ability to induce an antigen-specific Th1-biased response, conferring protective immunity and imparting significant protection against *U. urealyticum* in a mouse model. This response inhibits the pathogenesis of reproductive tract *U. urealyticum* challenges by decreasing the burden of the pathogen and eliminating it from the host. IFN-γ- and TNF-α-producing CD4^+^ Th1 cells may be crucial for this protective immune response. These results preliminarily indicate that GrpE is a potential antigen target for the development of future multi-antigenic vaccines against *U. urealyticum* and provide novel and important information on *U. urealyticum* pathogenic mechanisms.

## Data Availability Statement

The datasets generated for this study are available on request to the corresponding author.

## Ethics Statement

The animal study was reviewed and approved by the Animal Welfare Committee of the University of South China.

## Author Contributions

YT: data preparation and interpretation, animal experiment, and writing the manuscript. FG: purification of recombinant protein. AL: flow detection. JX: modifying the manuscript. PL: western blotting experiments. WT: pathological analysis. RL: participation in language editing and intellectual contribution throughout the study. GD: intellectual contribution throughout the study and interpretation of data. All authors reviewed the manuscript and read and approved the final manuscript.

## Conflict of Interest

The authors declare that the research was conducted in the absence of any commercial or financial relationships that could be construed as a potential conflict of interest.

## References

[1] Glass JI Lefkowitz EJ Glass JS Heiner CR Chen EY Cassell GH. The complete sequence of the mucosal pathogen *Ureaplasma urealyticum*. Nature. (2000) 407:757–62. 10.1038/35037619 11048724

[2] Silwedel C Haarmann A Fehrholz M Claus H Speer CP Glaser KJJ. More than just inflammation: Ureaplasma species induce apoptosis in human brain microvascular endothelial cells. J Neuroinflamm. (2019) 16:38. 10.1186/s12974-019-1413-8 30764830 PMC6374915

[3] Radonic A Kovacevic V Markotic A Skerk V Turcic P Skerk V. The clinical significance of *Ureaplasma urealyticum* in chronic prostatitis. J Chemother. (2009) 21:465–6. 10.1179/joc.2009.21.4.465 19622472

[4] Deetjen P Maurer C Rank A Berlis A Schubert S Hoffmann R. Brain abscess caused by *Ureaplasma urealyticum* in an adult patient. J Clin Microbiol. (2014) 52:695–8. 10.1128/JCM.02990-13 24478517 PMC3911303

[5] Wang X Karau MJ Greenwood-Quaintance KE Block DR Mandrekar JN Cunningham SA. *Ureaplasma urealyticum* causes hyperammonemia in an experimental immunocompromised murine model. PLoS ONE. (2016) 11:e0161214. 10.1371/journal.pone.0161214 27537683 PMC4990232

[6] Beeton ML Payne MS Jones L. The role of ureaplasma spp. in the development of nongonococcal urethritis and infertility among men. Clin Microbiol Rev. (2019) 32:e00137-18. 10.1128/CMR.00137-18 31270127 PMC6750135

[7] Sweeney EL Dando SJ Kallapur SG Knox C. L. The human ureaplasma species as causative agents of chorioamnionitis. Clin Microbiol Rev. (2017) 30:349. 10.1128/CMR.00091-16 27974410 PMC5217797

[8] Rittenschober-Bohm J Waldhoer T Schulz SM Stihsen B Pimpel B Goeral K. First trimester vaginal ureaplasma biovar colonization and preterm birth: results of a prospective multicenter study. Neonatology. (2018) 113:1–6. 10.1159/000480065 28934751

[9] Arango Duque G Descoteaux A. Macrophage cytokines: involvement in immunity and infectious diseases. Front Immunol. (2014) 5:491. 10.3389/fimmu.2014.00491 25339958 PMC4188125

[10] Glaser K Silwedel C Fehrholz M Waaga-Gasser AM Henrich B Claus H. Ureaplasma species differentially modulate pro- and anti-inflammatory cytokine responses in newborn and adult human monocytes pushing the state toward pro-inflammation. Front Cell Infect Microbiol. (2017) 7:484. 10.3389/fcimb.2017.00484 29234642 PMC5712342

[11] Shah SS. Ureaplasma urealyticum. Principles & Practice of Pediatric Infectious Diseases. New York, NY: Elsevier Churchill Livingstone (2012). p. 1000–02.

[12] Zhang Y Hua C Li SL. The relationship between the biovars and the antimicrobial resistance of *Ureaplasma urealyticum* in female patients with urogenital infections. J Clin Lab Anal. (2018) 32:e22211. 10.1002/jcla.22211 28345794 PMC6816965

[13] Wilkinson KA Stewart GR Newton SM Vordermeier HM Wain JR Murphy HN. Infection biology of a novel alpha-crystallin of Mycobacterium tuberculosis: Acr2. J Immunol. (2005) 174:4237–43. 10.4049/jimmunol.174.7.4237 15778386

[14] Shekhawat SD Purohit HJ Taori GM Daginawala HF Kashyap RS. Evaluation of heat shock proteins for discriminating between latent tuberculosis infection and active tuberculosis: a preliminary report. J Infect Public Health. (2016) 9:143–52. 10.1016/j.jiph.2015.07.003 26300163

[15] Harmala LA Ingulli EG Curtsinger JM Lucido MM Schmidt CS Weigel BJ. The adjuvant effects of Mycobacterium tuberculosis heat shock protein 70 result from the rapid and prolonged activation of antigen-specific CD8+ T cells in vivo. J Immunol. (2002) 169:5622–9. 10.4049/jimmunol.169.10.5622 12421941

[16] Bracher A Verghese J. GrpE, Hsp110/Grp170, HspBP1/Sil1 and BAG domain proteins: nucleotide exchange factors for Hsp70 molecular chaperones. Subcell Biochem. (2015) 78:1–33. 10.1007/978-3-319-11731-7_1 25487014

[17] Harrison C. GrpE, a nucleotide exchange factor for DnaK. Cell Stress Chaperones. (2003) 8:218–24. 10.1379/1466-1268(2003)008<0218:GANEFF>2.0.CO;2 14984054 PMC514874

[18] Cayabyab MJ Macovei L Campos-Neto A. Current and novel approaches to vaccine development against tuberculosis. Front Cell Infect Microbiol. (2012) 2:154. 10.3389/fcimb.2012.00154 23230563 PMC3515764

[19] Aguilo N Gonzaloasensio J Alvarezarguedas S Marinova D Gomez AB Uranga S. Reactogenicity to major tuberculosis antigens absent in BCG is linked to improved protection against *Mycobacterium tuberculosis*. Nat Commun. (2017) 8:16085. 10.1038/ncomms16085 28706226 PMC5519979

[20] Lu C Zeng H Li Z Lei L Yeh IT Wu Y. Protective immunity against mouse upper genital tract pathology correlates with high IFNγ but low IL-17 T cell and anti-secretion protein antibody responses induced by replicating chlamydial organisms in the airway. Vaccine. (2012) 30:475–85. 10.1016/j.vaccine.2011.10.059 22079265 PMC3246108

[21] Lu C Peng B Li Z Lei L Li Z Chen L. Induction of protective immunity against *Chlamydia muridarum* intravaginal infection with the chlamydial immunodominant antigen macrophage infectivity potentiator. Microbes Infect. (2013) 15:329–38. 10.1016/j.micinf.2013.02.001 23416214 PMC4218745

[22] Li Y Zheng K Tan Y Wen Y Wang C Chen Q. A recombinant multi-epitope peptide vaccine based on MOMP and CPSIT_p6 protein protects against *Chlamydia psittaci* lung infection. Appl Microbiol Biotechnol. (2019) 103:941–52. 10.1007/s00253-018-9513-4 30467705

[23] Fairley SJ Singh SR Yilma AN Waffo AB Subbarayan P Dixit S. Chlamydia trachomatis recombinant MOMP encapsulated in PLGA nanoparticles triggers primarily T helper 1 cellular and antibody immune responses in mice: a desirable candidate nanovaccine. Int J Nanomed. (2013) 8:2085–99. 10.2147/IJN.S44155 23785233 PMC3682632

[24] Kwon KW Kim WS Kim H Han SJ Hahn MY Lee JS. Novel vaccine potential of Rv3131, a DosR regulon-encoded putative nitroreductase, against hyper-virulent Mycobacterium tuberculosis strain K. Sci Rep. (2017) 7:44151. 10.1038/srep44151 28272457 PMC5341159

[25] Guo F Liu Y Zhang C Wang Q Wang L Gao Y. Prompt and robust humoral immunity elicited by a conjugated chimeric malaria antigen with a truncated flagellin. Bioconjug Chem. (2018) 29:761–70. 10.1021/acs.bioconjchem.7b00320 28795800

[26] Conrad TA Gong S Yang Z Matulich P Keck J Beltrami N. The chromosome-encoded hypothetical protein TC0668 is an upper genital tract pathogenicity factor of *Chlamydia muridarum*. Infect Immun. (2016) 84:467–79. 10.1128/IAI.01171-15 26597987 PMC4730586

[27] Yoder BA Coalson JJ Winter VT Siler-Khodr T Duffy LB Cassell GH. Effects of antenatal colonization with *ureaplasma urealyticum* on pulmonary disease in the immature baboon. Pediatr Res. (2003) 54:797–807. 10.1203/01.PDR.0000091284.84322.16 12930907

[28] Normann E Lacaze-Masmonteil T Eaton F Schwendimann L Gressens P Thebaud B. A novel mouse model of Ureaplasma-induced perinatal inflammation: effects on lung and brain injury. Pediatr Res. (2009) 65:430–6. 10.1203/PDR.0b013e31819984ce 19127208

[29] Zhu GX Lu C Chen CJ Feng PY Ma H Lu RB. Pathogenicity of *Ureaplasma urealyticum* and *Ureaplasma parvum* in the lower genital tract of female BALB/c mice. Can J Microbiol. (2011) 57:987–92. 10.1139/w11-098 22106821

[30] Kong F Ma Z James G Gordon S Gilbert G. L. Species identification and subtyping of *Ureaplasma parvum* and *Ureaplasma urealyticum* using PCR-based assays. J Clin Microbiol. (2000) 38:1175–9. 10.1128/JCM.38.3.1175-1179.2000 10699016 PMC86368

[31] Xiao L Glass JI Paralanov V Yooseph S Cassell GH Duffy LB. Detection and characterization of human Ureaplasma species and serovars by real-time PCR. J Clin Microbiol. (2010) 48:2715–23. 10.1128/JCM.01877-09 20554828 PMC2916572

[32] Vancutsem E Soetens O Breugelmans M Foulon W Naessens A. Modified real-time PCR for detecting, differentiating, and quantifying *Ureaplasma urealyticum* and *Ureaplasma parvum*. J Mol Diagn. (2011) 13:206–12. 10.1016/j.jmoldx.2010.10.007 21354056 PMC3128564

[33] Mallard K Schopfer K Bodmer T. Development of real-time PCR for the differential detection and quantification of *Ureaplasma urealyticum* and *Ureaplasma parvum*. J Microbiol Methods. (2005) 60:13–9. 10.1016/j.mimet.2004.08.005 15567220

[34] Yi J Yoon BH Kim EC. Detection and biovar discrimination of *Ureaplasma urealyticum* by real-time PCR. Mol Cell Probes. (2005) 19:255–60. 10.1016/j.mcp.2005.04.002 16005182

[35] Cao X Jiang Z Wang Y Gong R Zhang C. Two multiplex real-time TaqMan polymerase chain reaction systems for simultaneous detecting and serotyping of *Ureaplasma parvum*. Diagn Microbiol Infect Dis. (2007) 59:109–11. 10.1016/j.diagmicrobio.2007.04.014 17574785

[36] Yuanyuan S Qin S Rongrong X Yujing G Chengbin P Jianjun M. Reference gene selection for real-time quantitative PCR analysis on ovarian cryopreservation by vitrification in mice. J Assist Reprod Genet. (2015) 32:1277–84. 10.1007/s10815-015-0503-5 26115720 PMC4554379

[37] O'Meara CP Armitage CW Harvie MC Timms P Lycke NY Beagley KW. Immunization with a MOMP-based vaccine protects mice against a pulmonary Chlamydia challenge and identifies a disconnection between infection and pathology. PLoS ONE. (2013) 8:e61962. 10.1371/journal.pone.0061962 23613984 PMC3628704

[38] Barry MA Johnston SA. Biological features of genetic immunization. Vaccine. (1997) 15:788–91. 10.1016/S0264-410X(96)00265-4 9234514

[39] Uyeda S Sharmin T Satho T Irie K Watanabe M Hosokawa M. Enhancement and regulation effect of myrcene on antibody response in immunization with ovalbumin and Ag85B in mice. Asian Pac J Allergy Immunol. (2016) 34:314–23. 10.12932/AP0734 27543726

[40] Marks E Verolin M Stensson A Lycke N. Differential CD28 and inducible costimulatory molecule signaling requirements for protective CD4+ T-cell-mediated immunity against genital tract *Chlamydia trachomatis* infection. Infect Immun. (2007) 75:4638–47. 10.1128/IAI.00465-07 17635872 PMC1951167

[41] Morrison SG Su H Caldwell HD Morrison RP Immunity to murine Chlamydia trachomatis genital tract reinfection involves B cells and CD4(+) T cells but not CD8(+) T cells. J Infect Immun. (2000) 68:6979–87. 10.1128/IAI.68.12.6979-6987.2000 PMC97807 11083822

[42] Beck L Spiegelberg HL The polyclonal and antigen-specific IgE and IgG subclass response of mice injected with ovalbumin in alum or complete Freund's adjuvant. Cell Immunol. (1989) 123:1–8. 10.1016/0008-8749(89)90263-3 2776217

[43] Fernando GJ Stewart TJ Tindle RW Frazer IH. Vaccine-induced Th1-type responses are dominant over Th2-type responses in the short term whereas pre-existing Th2 responses are dominant in the longer term. Scand J Immunol. (1998) 47:459–65. 10.1046/j.1365-3083.1998.00327.x 9627130

[44] Marciani DJ. Vaccine adjuvants: role and mechanisms of action in vaccine immunogenicity. Drug Discov Today. (2003) 8:934–43. 10.1016/S1359-6446(03)02864-2 14554157

[45] Hwang SH Shin MS Yoon TJ Shin KS Immunoadjuvant activity in mice of polysaccharides isolated from the leaves of Panax ginseng C.A. Meyer. Int J Biol Macromol. (2018) 107(Pt B):2695–700. 10.1016/j.ijbiomac.2017.10.160 29107141

[46] Liang M Wen Y Ran O Chen L Wang C Li L. Protective immunity induced by recombinant protein CPSIT_p8 of Chlamydia psittaci. Appl Microbiol Biotechnol. (2016) 100:6385–93. 10.1007/s00253-016-7494-8 27052378

[47] Kim WS Kim JS Kim HM Kwon KW Eum SY Shin SJ. Comparison of immunogenicity and vaccine efficacy between heat-shock proteins, HSP70 and GrpE, in the DnaK operon of *Mycobacterium tuberculosis*. Sci Rep. (2018) 8:14411. 10.1038/s41598-018-32799-z 30258084 PMC6158166

[48] Furr PM Taylor-Robinson D. The establishment and persistence of *Ureaplasma urealyticum* in oestradiol-treated female mice. J Med Microbiol. (1989) 29:111–4. 10.1099/00222615-29-2-111 2733020

[49] von Chamier M Allam A Brown MB Reinhard MK Reyes L. Host genetic background impacts disease outcome during intrauterine infection with Ureaplasma parvum. PLoS ONE. (2012) 7:e44047. 10.1371/journal.pone.0044047 22952869 PMC3430619

[50] Bastek JA Gomez LM Elovitz MA. The role of inflammation and infection in preterm birth. Clin Perinatol. (2011) 38:385–406. 10.1016/j.clp.2011.06.003 21890015

[51] Senthamaraikannan P Presicce P Rueda CM Maneenil G Schmidt AF Miller LA. Intra-amniotic *Ureaplasma parvum*-induced maternal and fetal inflammation and immune responses in rhesus macaques. J Infect Dis. (2016) 214:1597–604. 10.1093/infdis/jiw408 27601620 PMC6392471

[52] Viscardi RM Kaplan J Lovchik JC He JR Hester L Rao S. Characterization of a murine model of *Ureaplasma urealyticum* pneumonia. Infect Immun. (2002) 70:5721–9. 10.1128/IAI.70.10.5721-5729.2002 12228302 PMC128302

[53] Shan P Lu Z Ye L Fang Y Tan S Xuan G. Effect of tripterygium wilfordii polyglycoside on experimental prostatitis caused by *Ureaplasma Urealyticum* in rats. Med Sci Monit. (2016) 22:3722–6. 10.12659/MSM.897360 27743513 PMC5070633

[54] O'Meara CP Andrew DW Beagley KW. The mouse model of Chlamydia genital tract infection: a review of infection, disease, immunity and vaccine development. Curr Mol Med. (2014) 14:396–421. 10.2174/15665240113136660078 24102506

[55] Qie YQ Wang JL Liu W Shen H Chen JZ Zhu BD. More vaccine efficacy studies on the recombinant Bacille Calmette-Guerin co-expressing Ag85B, Mpt64 and Mtb8.4. Scand J Immunol. (2009) 69:342–50. 10.1111/j.1365-3083.2009.02231.x 19284499

[56] Bourigault ML Vacher R Rose S Olleros ML Janssens JP Quesniaux VF. Tumor necrosis factor neutralization combined with chemotherapy enhances Mycobacterium tuberculosis clearance and reduces lung pathology. Am J Clin Exp Immunol. (2013) 2:124−34. 23885330 PMC3714199

[57] Armand M Chhor V de Lauzanne A Guerin-El Khourouj V Pedron B Jeljeli M. Cytokine responses to quantiferon peptides in pediatric tuberculosis: a pilot study. J Infect. (2014) 68:62–70. 10.1016/j.jinf.2013.08.005 23954615

[58] Hartley MG Green M Choules G Rogers D Rees DGC Newstead S. Protection afforded by heat shock protein 60 from *Francisella tularensis* is due to copurified lipopolysaccharide. Microb Immun Vacc. (2004) 72:4109–13. 10.1128/IAI.72.7.4109-4113.2004 15213156 PMC427437

