# Supplementary material for: GrpE Immunization Protects Against Ureaplasma urealyticum Infection in BALB/C Mice
Source: Front Immunol. 2020 Jul 31;11:1495. doi: 10.3389/fimmu.2020.01495 (PMC7411329; doi:10.3389/fimmu.2020.01495)
Supplement: Supplementary file 1 [file Data_Sheet_1.docx]

Supplementary Material

## Supplementary Figures


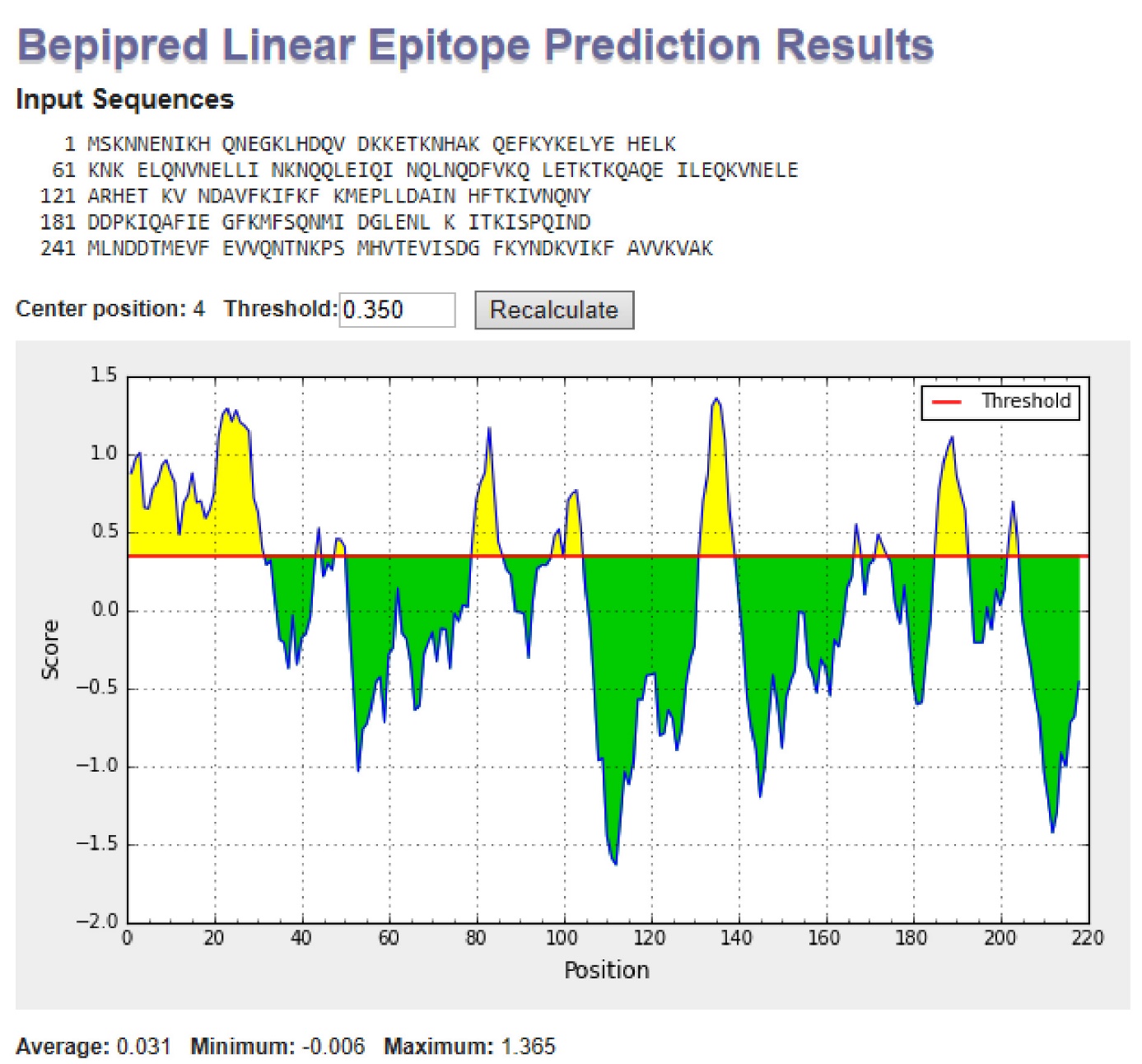


**Supplementary Figure 1.** Bioinformatics software predicts GrpE protein B cell epitopes in *U. urealyticum* (yellow)


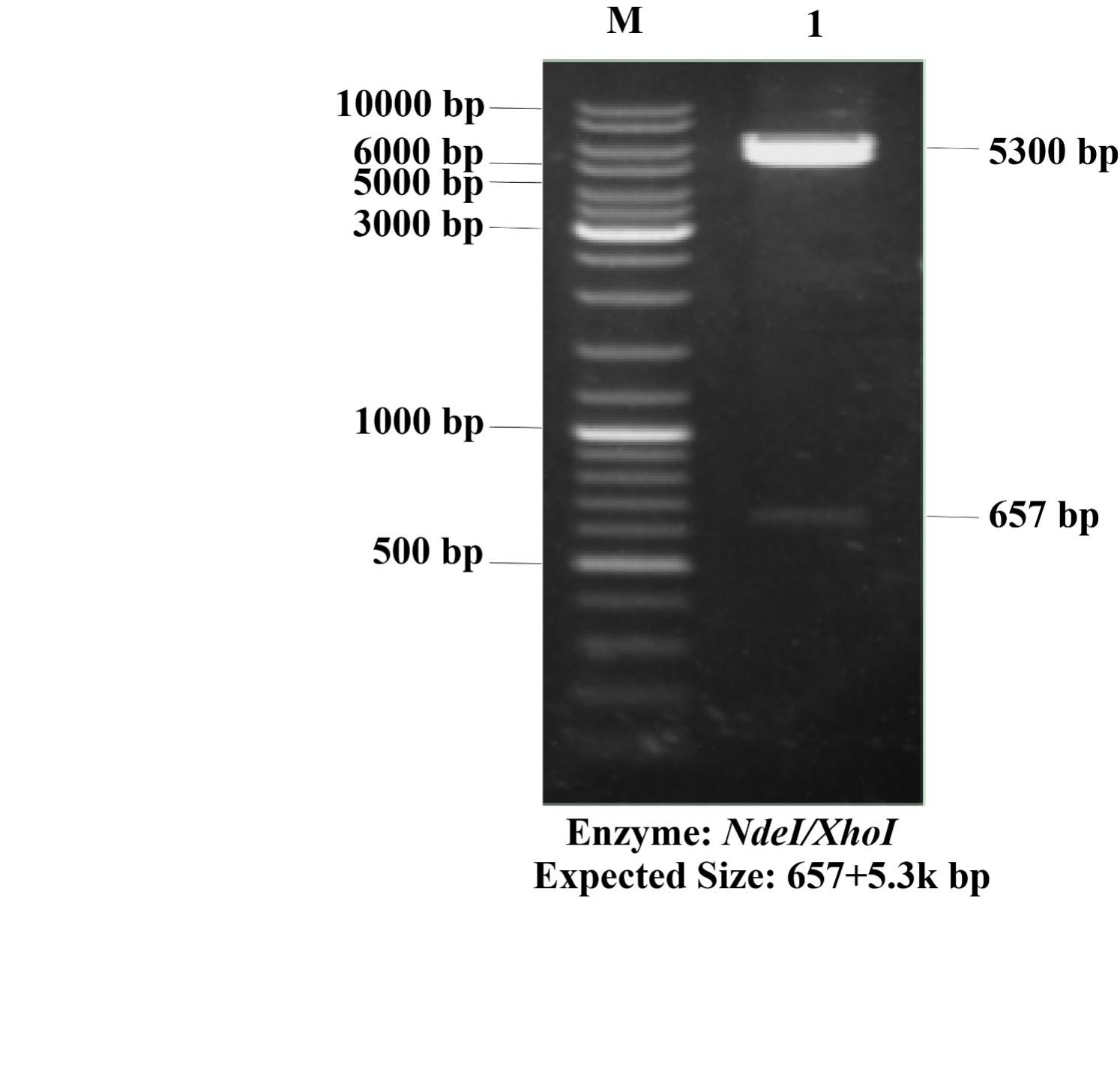


**Supplementary Figure 2.** Enzyme digestion results. PCR successfully amplified the expected GrpE gene band (657 bp) and cloned in *E. coli* BL21 strain with *NdeI* and *XhoI* restriction sites. M: marker, 1: The vector of pET-28a and the gene of GrpE


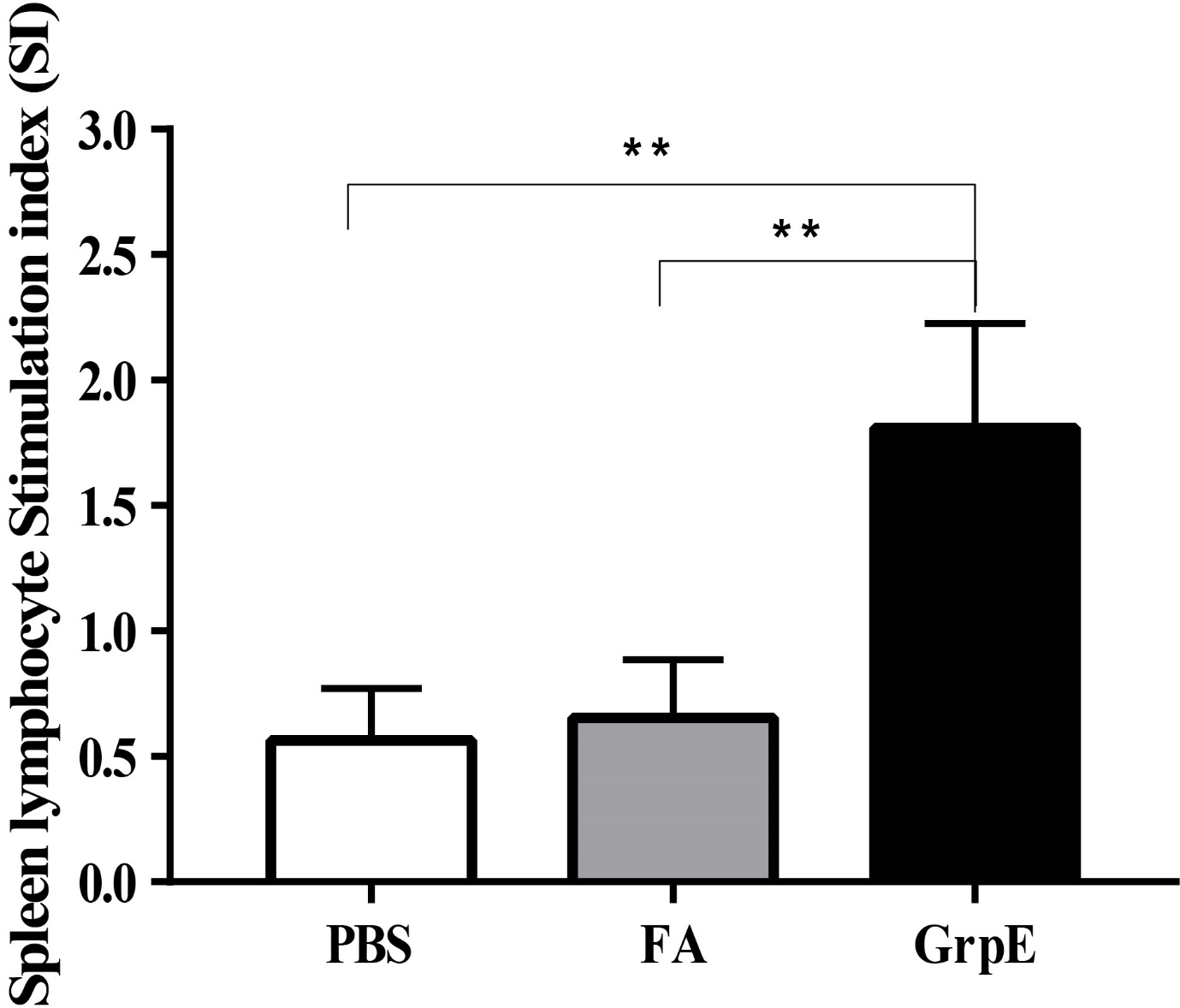


**Supplementary Figure 3.** Effect of the recombinant GrpE on splenic lymphocytes proliferation in mice. After 14 day of last immunization, the splenocytes (1×10^6^ cells/well) were Cultured in the presence of 10 ug of GrpE for 44 h in a CO_2_ incubator at 37◦C, Then add CCK-8, 20 uL / well, and After 4 h, the supernatant was collected for detection of spleen lymphocyte stimulation index, (**PBS and FA group vs. immunized , *p < 0.01*).

**Supplementary Table 1.** The Concentration of ureaplasma urealyticum from mouse vaginal and cervical secretions in solid medium (n=6 each group).

Vaginal discharge

| days | PBS | FA | GrpE |
| --- | --- | --- | --- |
| 7 d | 13.10±1.38 | 11.74±0.18 | 7.18±0.05 |
| 14 d | 11.92±1.14 | 9.43±1.23 | 5.23±0.67 |
| 21 d | 5.96±1.02 | 4.67±0.86 | 2.68±0.54 |

Cervical secretions

| days | PBS | FA | GrpE |
| --- | --- | --- | --- |
| 7 d | 10.58±0.92 | 9.82±0.76 | 5.26±0.21 |
| 14 d | 8.62±0.67 | 6.54±0.87 | 3.21±0.32 |
| 21 d | 4.35±0.43 | 3.21±0.65 | 1.20±0.21 |
